# Evaluation of herbal cannabis characteristics by medical users: a randomized trial

**DOI:** 10.1186/1477-7517-3-32

**Published:** 2006-11-13

**Authors:** Mark A Ware, Thierry Ducruet, Ann R Robinson

**Affiliations:** 1 Departments of Anesthesia and Family Medicine, McGill University, Montreal, Quebec, Canada; 2 Boreal Primum Inc., 913 Cherrier, Montreal, Quebec, Canada

## Abstract

**Background:**

Cannabis, in herbal form, is widely used as self-medication by patients with diseases such as HIV/AIDS and multiple sclerosis suffering from symptoms including pain, muscle spasticity, stress and insomnia. Valid clinical studies of herbal cannabis require a product which is acceptable to patients in order to maximize adherence to study protocols.

**Methods:**

We conducted a randomized controlled crossover trial of 4 different herbal cannabis preparations among 8 experienced and authorized cannabis users with chronic pain. Preparations were varied with respect to grind size, THC content and humidity. Subjects received each preparation on a separate day and prepared the drug in their usual way in a dedicated and licensed clinical facility. They were asked to evaluate the products based on appearance (smell, colour, humidity, grind size, ease of preparation and overall appearance) and smoking characteristics (burn rate, hotness, harshness and taste). Five-point Likert scores were assigned to each characteristic. Scores were compared between preparations using ANOVA.

**Results:**

Seven subjects completed the study, and the product with highest THC content (12%), highest humidity (14%) and largest grind size (10 mm) was rated highest overall. Significant differences were noted between preparations on overall appearance and colour (p = 0.003).

**Discussion:**

While the small size of the study precludes broad conclusions, the study shows that medical cannabis users can appreciate differences in herbal product. A more acceptable cannabis product may increase recruitment and retention in clinical studies of medical cannabis.

## Background

It is now well-recognized that *Cannabis sativa *(marijuana, weed, pot) is being used by patients with chronic debilitating diseases such as HIV/AIDS [1], chronic non-cancer pain [2], epilepsy [3], multiple sclerosis [4] and amyotrophic lateral sclerosis [5] for the management of symptoms such as pain, nausea, poor sleep, anxiety and stress. Since 2002, cannabis for medical use has been produced by Prairie Plant Systems Inc (PPS) under license to Health Canada [6]. Cannabis is cultivated for use in clinical trials, open-label safety studies [7], and for distribution to persons authorized under the Medical Marihuana Access Regulations (MMAR) to use marihuana for medical purposes [8]. A number of authorized persons using this product initially reported concerns about the product, include the dryness, grind size (defined as the size of the particles after grinding raw herbal material), and tetrahydrocannabinol (THC) content of the cannabis material [9].

To improve the acceptability of the cannabis product provided to patients and to facilitate recruitment and retention of research subjects in clinical trials, Health Canada and PPS explored mechanisms to vary herbal cannabis products with varying levels of humidity, THC content, and grind size. This study was conducted to evaluate several herbal cannabis presentations in which dryness, grind size and THC content were varied. The aims of the study were to determine experienced users' preference for cannabis products, and to determine whether there was any consistent pattern towards a preferred product.

## Methods

A randomized, double-blind study of cannabis products was undertaken over a six week period in June-July 2004. Subjects were all current holders of valid authorizations to possess cannabis under the Medical Marijuana Access Regulations, and were currently using cannabis for medical purposes. Subjects agreed not to drive to or from their scheduled appointments.

Four (4) different PPS preparations of *Cannabis sativa *were evaluated; an identical species was used for all preparations. THC content was varied by blending the flowering heads with leaves from lower down the plant. The product was milled using a dry conical mill (Quadro^® ^Comill^®^, Waterloo, Ontario), with particle size varied using screens with circular holes of 5 or 10 mm diameter. Cannabis preparations were packaged at PPS in 30 gram bags and labeled as Presentation # 1 to Presentation # 4 prior to shipment to the site pharmacy. The site pharmacist weighed 1 g quantities of each preparation (LP 3200D scale, Sartorius, Canada). The drug was repackaged in plastic vials closed with a soft plastic/rubber top, which were then dispensed to the study nurse as needed on the days of use. The product was weighed and transferred from the original package to the dispensing container within 5 minutes, and the ambient temperature and humidity conditions in the pharmacy were recorded at the time of preparation (Digital Hygrometer DHM-010, Davidoff, Canada). The original package was resealed immediately after weighing the required amount of product.

The characteristics of the products were determined using a specially designed questionnaire. Items consisted of 5-point Likert scales and included physical characteristics (smell (0: very unpleasant; 4: very pleasant), humidity (0: unacceptably dry; 4: optimum humidity), appearance (0: looks very bad; 4: looks excellent), particle size (0: very poor, 4: excellent), colour (0: very poor, 4: excellent) and ease of preparation (0: very hard to use; 4: very easy to use) and smoking characteristics (hotness (0: very hot; 4: very cool), harshness (0: very harsh; 4: very smooth), burn rate (0: burns too fast; 4: burns just right), and taste (0: worse possible taste; 4: best possible taste). An overall comparison to usual cannabis was made (0: much worse than usual cannabis; 4: much better that usual cannabis). A global assessment was performed on the final day at which subjects were asked to rank the products assessed in order of preference (0: worse; 4: best). Subjects were not asked about clinical parameters such as efficacy.

Eligible subjects were given a scheduled series of 4 appointments. Informed consent was signed at the first appointment. The subjects were randomly assigned to test one cannabis preparation per day over the four days. They were asked to assess physical characteristics of the drug prior to use. They were asked to prepare and use the cannabis in the manner to which they were accustomed. Within five minutes of use they were asked to comment on the harshness, hotness, burn rate and comparison with usual cannabis. Subjects remained in the cannabis laboratory for one hour after use before being allowed to return home by taxi. On the evening after each clinic visit the subjects were contacted at home by the research nurse to determine if they had additional comments or concerns regarding the product they evaluated that day.

It was estimated that recruiting 8 subjects would allow a basic and preliminary assessment of trends in perceptions of differences between product characteristics. This number of subjects was also feasible to recruit within the time period of the study. The data were double entered and validated in a dedicated and secure database. Data were entered without identifiers to protect confidentiality. Cannabis preparations were ranked according to user preferences on each item. All items were rated equally. A total rank score for each preparation was assigned. Ranks according to individual physical and smoking characteristics were examined. All statistical analyses were done using SAS (SAS Institute, North Carolina). Means, frequencies and a multiple analysis of variance (ANOVA) for cross-over design (proc mixed) were calculated to assess the comparison between products. Period effect was analyzed independently. Due to the small sample-size, no adjustments were made for multiple comparisons.

The study was conducted at a dedicated cannabis research laboratory at the Montreal General Hospital in the McGill University Health Centre (MUHC). The study was approved by the Research Ethics Committee of the McGill University Health Centre.

## Results

Eight subjects were recruited for the study. No subject refused to participate, but one subject did not attend for the final visit and was excluded from the final analysis. Therefore the final report is based on seven subjects who completed the study. Inclusion of the eighth subject in a limited analysis did not alter the overall direction of the results. The period effect could not be assessed in the main model because one subject missed one visit; however no overall differences were found on overall comparison of the four time periods.

There were 5 males and 2 females, and the mean age was 47 years (range 40–54 y). Diagnoses were varied and included peripheral neuropathic pain (4 subjects), multiple sclerosis (2 subjects), and HIV/AIDS (1 subject).

The overall results based on the Likert scores for each characteristic and the overall scores are shown in Table 1. Product 1 (10.6% THC, 14.4% humidity, 10 mm grind size) was the most well rated product, and significant differences were noted between products overall (p = 0.03), and for physical characteristics including general appearance (p = 0.03) and colour (p = 0.03).

**Table 1:** Evaluation of four cannabis preparations by physical and smoking characteristics*

**Characteristic**	**Product**				***p*-value**	**Contrasts**
	**1**	**2**	**3**	**4**		
*Cannabis preparation*						
THC (g%)	10.6	10.6	6.6	9.6		
Humidity (%)	14.4	12	11	11		
Drying time (days)	2	4	4	4		
Grind size (mm)	10	5	5	10		
*Physical*						
Smell	2.71	3.00	2.57	2.43	0.21	
Humidity	1.71	1.86	1.71	1.14	0.28	
General appearance	2.71	2.29	1.43	1.86	0.03	1>3,4
Ease of preparation	3.29	3.14	3.29	2.86	0.55	
Colour	2.71	2.71	2.00	2.43	0.03	1,2>3
Particle size	2.86	2.43	1.57	1.86	0.06	
*Smoking*						
Hotness	1.86	1.86	2.14	1.86	0.81	
Harshness	2.14	2.14	1.43	1.71	0.53	
Burn rate	2.57	2.29	1.86	2.00	0.55	
Taste	2.00	2.14	1.29	2.00	0.41	
**TOTAL SCORE**	**24.57**	**23.86**	**19.29**	**20.14**	**0.03**	**1,2>3,4**

*Characteristic scores based on 5-point Likert scales (0–4). Higher scores reflect better ratings for each characteristic. See text for details.

Of the 28 different assessments (7 subjects using 4 products), 18 were performed using joints and 10 using pipes. Most assessments (93%) were done with subjects reporting using the same amount of cannabis as usual, and 16/18 stated no problems in preparing their joints. Product 4 received a poor rating in terms of problems rolling it by 2 subjects.

### Physical characteristics

Product 2 was treated as having the best smell with 6 subjects rating it pleasant or very pleasant. Product 2 was also rated as the best humidity with 6 subjects rating it acceptable. In general appearance, product 1 was superior; 4 subjects rated product 3 as 'looks bad'. Products 1, 2 and 3 were rated similar in terms of ease of preparation, although all 7 subjects rated products 1 and 2 as easy or very easy to use. Products 1 and 2 were rated equally in terms of colour. Product 1 was rated best in terms of particle size with 5 subjects rating particle size as good or excellent.

### Smoking characteristics

Product 3 was rated most 'cool' overall. Product 2 was rated highest in terms of harshness with 6 subjects rating it as moderate or smooth. Product 1 was rated as having the best burn rate (1 subject stated it 'burned just right'), while each of products 2, 3 and 4 were rated by one subject each as burning too fast. Product 2 was rated as having the best overall taste.

### Global assessments

Fourteen out of 28 (50%) subjects rated products 1, 3 and 4 as 'worse than their usual cannabis', 11 assessments were the 'same as usual cannabis' (4 of which were for product 2). Only 3 assessments were 'better than usual cannabis' (products 1, 2 and 4). Globally, product 1 received the highest score in terms of all the product characteristics measured (Table 2). In this overall analysis, product 1 was superior to products 3 and 4, and product 2 was superior to product 3.

Of the products tested, over half (4/7) of those using products 1 and 2 would use it on a regular basis, and over half would not use products 3 (5/7) and 4 (5/6) on a regular basis. In terms of overall satisfaction, 3 subjects rated product 1 as good or excellent and 2 subjects rated the other products as good or excellent. Five subjects rated product 3 as poor, and one rated product 2 as very poor.

### Ambient humidity and temperature

The drug samples were prepared the day before the visit for the first 5 patients, resulting in the drug being in the new container for 16 to 20 hours. For the last 3 patients, the drug spent from 6 to 17 days in the new container. Temperature and humidity measurements were taken in the pharmacy on 14 days over the 28 day period. Over this period, the mean room temperature was 22.4°C (SD 0.23), and the mean ambient humidity was 46% (SD 4.4).

## Discussion

To our knowledge, this is the first ever evaluation of medical cannabis products for physical and smoking characteristics by authorized patients. We have shown that subjects may appreciate differences between cannabis preparations on the basis of physical characteristics of the herbal material, specifically general appearance and colour. We did not show differences in individual smoking characteristics, but overall impressions confirmed that subjects favoured higher THC content, higher humidity and larger grind size.

The results of this study must be interpreted with considerable caution as there are many limitations to the data obtained. The small sample size reduces the power of the study to reach definitive conclusions about patient preference. The detected differences between products could have arisen by chance, or may have been influenced by other factors such as the use of different modes of administration (pipes and joints). Further study should limit the modes of administration to reduce confounding by these factors.

The effect of room air on the humidity levels of the product is a factor which may affect the validity of the final results. The average ambient humidity at the time of product preparation was well above that of the original product specifications (46% versus 10–15%), and could have raised the humidity of the product during the storage period prior to use. This would have the effect of reducing or eliminating the potential differences between products on humidity-based assessments. This may partially explain why differences in physical characteristics such as colour, particle size and general appearance were detected (Table 2), while the hotness, harshness and burn rate appeared to be rated similarly between products.

Subjects' evaluations of smoking characteristics of the samples immediately after use may have been influenced by the psychoactive effects of cannabis. This study recruited experienced medicinal cannabis users who would likely evaluate any cannabis product under similar conditions ('try it and see') so we feel our approach is pragmatic and relevant.

The subjects and investigators were initially blind to the characteristics of the allocated products, but the ability of the subjects to differentiate the products suggests that the blinded condition was compromised. The investigators (study nurse or physician) did not evaluate their own ability to differentiate the products

**Figure 1:**
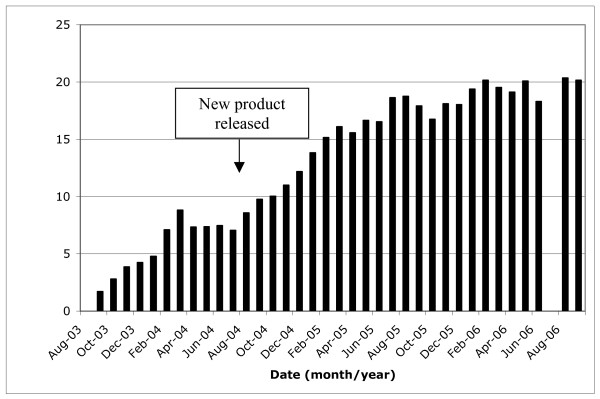
Percentage of authorized users obtaining herbal cannabis from Health Canada (data to Sept 2006)^10^.

In spite of these potential limitations, this randomized double-blind study has found that two of these products (products 1 and 2) could be appreciated differently from the other two, in terms of their physical and smoking characteristics. Product 3, which was a 10% THC blend (expiry date June 2004) which had been originally shipped by Health Canada to authorized patients was rated poorly by the subjects in this study, suggesting that the product could be improved by changing physical characteristics such as blending, particle size and humidity. These changes may result in improved patient satisfaction with the product, which may in turn increase the number of patients willing to use the Health Canada product and improve compliance in long term studies using the product. The study results support a decision by Health Canada, made prior to the study in May 2004, to distribute a product made only of flowering head material, taking into account preliminary reports from authorized users, with larger grind size, higher humidity and higher THC content. A review of Health Canada statistics [10] suggests that use of the Health Canada product increased since the new product was shipped in the summer of 2004 (Figure 1). An initial delay in uptake may have been due to media reports from disgruntled users about the poor quality of the product [11].

This study should ideally be repeated with larger numbers to validate differences between products. The reliability of the subjects' reports may also be validated by repeating the test with the same subjects and products to see if there is reliability between their assessments over time. Future studies of the effect of humidity could be done with subjects removing cannabis directly from an unopened original package prior to use rather than going through pharmacy dispensing. Methods of rehumidification of herbal material should be explored. Finally, this study design may also be used to detect differences between cannabis products with different cannabinoid profiles or phenotypic characteristics.

## Conclusion

We have shown that medical cannabis users may discriminate between cannabis preparations based on physical characteristics such as humidity, grind size, and smoking characteristics. The supply of a standardized herbal cannabis product within a legal medical access program needs to be guided by user's feedback to ensure compliance. Further work is required on other characteristics such as the profile of cannabinoids and other constituents.

## Authors' contributions

The study was designed and supervised by Dr. Mark Ware, who prepared the final report and who is responsible for the work. Study management, including coordination, data collection, data entry and statistical analysis, was provided under subcontract to Boreal Primum Inc.

## Competing interests

Dr Ware has received speakers fees, honoraria and grant support from companies developing cannabinoid products (AstraZeneca, Bayer, Cannasat, GW Pharma, Solvay and Valeant).
